# Progress in Surgery

**Published:** 1902-06-28

**Authors:** 


### Progress in Surgery.

(*Continued from page* 188.)

### OTOLOGY.

**The Indications for Operative Interference in Mastoid Suppuration.**—Wendell C. Phillips (New York)[7] has contributed a paper on this subject. Acute and chronic mastoid suppuration have to be considered. By acute suppurative involvement is meant an extension of the suppurative process from the middle ear into the mastoid antrum and cells during an acute attack of middle-ear suppuration. An acute mastoid involvement following chronic middle-ear suppuration is generally spoken of as chronic. The writer then alludes to the great risk there is of severe ear-complications following influenza, and suggests that more attention should be given to the cleansing of the nose and nasopharynx, so as to render the Eustachian tube less liable to an invasion of bacilli. In acute ear suppuration he advocates also that the nature of the bacteria in the pus should be ascertained, as those cases are the most likely to be complicated with mastoid involverment in which staphylococci and streptococci are present, especially if the streptococci are in excess. Blood examinations are recommended if infection of the lateral sinus is suspected. Pain is perhaps the most marked symptom. It usually comes on some time after the excruciating pain which precedes suppuration of the middle ear has passed away, and after ear discharge has been established. It is a dull heavy pain, not definitely localised like the earlier pain from the otitis, but diffused over the surface of the temporal bone, especially behind and above the pinna. There is with it a feeling of pressure and fulness over the entire parietal region. It may not be constant. Tenderness on pressure is another prominent symptom, and is of more significance if located high up just over the antrum. The tip of the mastoid may be tender when no disease is present. The temperature may be but little disturbed in adults. The facial expression is indicative of anxiety. The head usually hangs forward with an inclination to the healthy side. External periostitis must be looked upon rather as a complication than a symptom, and usually operation should be performed before it appears. The involvement of Shrapnell's membrane or the attic region, and bulging downwards of the posterior superior wall of the meatus are very characteristic of mastoid involvement. The existence of the latter sign, together with that of tenderness over the antrum, shows that an operation should be performed. Sometimes the very early symptoms may be relieved by a free incision of the drum membrane carried well through the attic region into the external canal. Local blood-letting, poultices, the ice-coil, and irrigation with hot sterile water used once or twice each hour are also useful. The patient should be kept in bed. When the above measures fail to abolish the symptoms of mastoid involvement an external operation should be performed without delay. In many cases it is a mistake to attempt to treat the disease by any other method. Early operation will usually be followed by perfect hearing. To make a Wilde incision only is strongly condemned, whether the bone exposed by the incision is healthy looking or not.

**Otitic Lateral Sinus Thrombosis.**—S. H. Law (Dublin)[8] reports a case of this successfully treated. The patient, a man nineteen years of age, was unwell for twenty days before the operation was performed. The earlier symptoms were headache, vomiting, and slight ear pain. On the fourth day the temperature was 102·6°, and there was some cough. The temperature became normal for a week and then rose again, and soreness in the neck developed. On the day of operation the symptoms were more serious. A severe rigor occurred with a temperature of 105·8°, there was some rusty sputum with the cough, and a whitlow developed on one finger. The operation consisted in tying the jugular vein in two places, then opening the mastoid antrum after the method of Schwartze, and evacuating pus, then exposing the lateral sinus by enlarging the antral opening backwards. An extra-dural collection of pus over the lateral sinus was thus cleared out and granulations and a slough seen on the outer wall of the sinus. The latter pulsated well and was not opened. The jugular vein was divided between the two ligatures. The temperature oscillated for ten days and then became normal. The membrana tympani finally almost regained its normal appearance, and the patient could hear whispers at two yards. Murrell and De Santi[9] relate a case of otitic pyæmia which ended fatally, and in which at the post-mortem no purulent invasion of the attic or antrum and no sinus infection was found. There were general purulent meningitis, a small quantity of pus in the tympanum, and some vegetations in the mitral valve. The chief symptoms during the illness, which caused death and which lasted about eight weeks, were noisy delirium, rigors, an abscess over the sacrum and in the right knee-joint. The occurrence of pyæmia from ear disease without implication of a cerebral sinus is considered by Leutert to be improbable and at best rare, and to have not yet been proven.

**Otitic Brain Abscess.**—Robert Lewis, jun. (New York)[10] reports an instructive case of this kind. The patient was a woman of 23, and had had purulent ear-discharge since childhood. Her illness began about 10 days before the brain abscess was opened. Her symptoms began with a cold and cessation of ear discharge, accompanied by tinnitus, ear pain, and headache. By the end of a week, after having had three severe rigors on three consecutive days, drowsiness, muttering delirium, and amnesic aphasia developed. There were signs of mastoiditis, but no tenderness in the neck, and no paralysis or optic neuritis. The aphasia was marked. She could not name the objects placed before her, but could correctly state the purpose for which they were used. At the operation the antrum was opened and emptied of foul discharge, and the lateral sinus exposed and found to be covered with pus and granulations. The greater part of the squamous portion of the temporal bone was removed. Everywhere the dura beneath it was covered with pus. An incision was made over a tense part of the dura covering the temporo-sphenoidal lobe, and an abscess cavity of the size of a large walnut evacuated. The aphasia completely disappeared in about a month. The author points out that the occurrence of such cases as this, secondary to chronic otorrhœa, generally mean that there has been culpable neglect in the treatment of acute otitis, the neglect resulting in the acute inflammation becoming chronic. He feels warranted in saying that every child born into the world should be examined a number of times during its early life for hypertrophy of the pharyngeal tonsil, and have it removed if found.

[7] American Jour. Med. Science, Dec. 1901, p. 790. [8] Brit. Med. Jour., Jan. 4, 1902, p. 17. [9] Brit. Med. Jour., Dec. 21, 1901, p. 1806. [10] Med. Rec., March 13, 1902, p. 452.

